# Two-stage revision of infected hip prosthesis after post-operative antibiotic therapy: An observational study

**DOI:** 10.1097/MD.0000000000032878

**Published:** 2023-02-10

**Authors:** Afshin Taheriazam, Amin Saeidinia

**Affiliations:** a Department of Orthopedics Surgery, Tehran Medical Sciences Branch, Islamic Azad University, Tehran, Iran; b Mashhad University of Medical Sciences, Mashhad, Iran.

**Keywords:** antibiotic, infection, periprosthetic infection, reoperation, total hip arthroplasty

## Abstract

Infection is a major threatening side effect after total hip arthroplasty (THA) that its management is so difficult and is accompanied by different complications. The aim of this study was to determine the outcomes of patients underwent 2-staged THA after a course of antibiotic therapy. It was an observational prospective study performed during 2009 and 2019. We managed 51 patients with infected THA using a method in which antibiotic prophylaxis was performed after THA. We followed the same protocol for treatment of patients included 2-staged revision: in first stage, removal of infected instruments were performed and insertion of a hand-made antibiotic-cement spacer was done until erythrocyte sedimentation rate and CRP were normalized. In second stage, an un-cemented prosthesis was re-implanted in femoral side and post-operative IV antibiotic were administered for a week. Patients were monitored for about 15 months. Data were analyzed. There were 3 patients developed recurrent infection required girdlestone due to the aging. One of them needed to remove implant and 2 other with 3 times of re-infection were treated by antibiotic therapy. Other 10 cases were treated first by re-changing the cement. The rate of successful treatment was 78.4% (40 of 51) after the primary surgery and antibiotic therapy. This rose to 92.1% (47 of 51) following more debridement and antibiotic therapy. The merging of staged surgical debridement, using spacer of cement-antibiotic and re-implant beside 1-week intravenous antibiotic therapy, leaded to appropriate early outcomes in this series.

## 1. Introduction

Using antibiotics has been changed in different aspects such as kind and family of antibiotics, sensitivity, and resistance pattern to various antibiotics.^[1]^ Profound infection followed by total hip arthroplasty (THA) is a destructive side effect, which encountered orthopedics in a challenge for better treatment planning.^[2–4]^ There are different approaches for treating infections consisted of 1-stage revision, long-term antibiotic suppression treatment, multiple debridement with implant conservation and 2-step revision.^[2–6]^ The most used technique for management of infections is staged re-implantation of prosthesis after systemic antibiotic therapy.^[7]^ Despite of lower rate of infection after primary THA (<1%),^[2,5,6,8–11]^ the rate of infection after 2-step THA is reported between 3.2% and 13%.^[12–15]^ Recent guidelines have mentioned the improvement in diagnostic approaches of pre-operative infections.^[16–19]^

There is no consensus for necessity of post-operative antibiotic therapy, currently.^[7]^ Despite of success in 1-step revision and debridement in selected cases,^[10,20]^ the standard preferred surgical technique in management of prosthetic hip infections (PHIs) is staged arthroplasty included removing all components beside debridement of infected tissue and antibiotic therapy followed by re-insertion of new prosthesis at final stage.^[21]^ The use of an antibiotic-loaded cement spacer (ALCS) has been changed to a frequent technique. Using ALCS can used for a temporary hip joint implant and also a technique for direct antibiotic delivery.^[3]^ The role of ALCSs in the treatment of PJI is 2-fold: mechanical and pharmaceutical. The ALCS fills the gap, which occurs after the removal of the infected prosthesis and surgical debridement. Therefore, it prevents extensive scarring, preserves the quality of the bone and the length of the limb and facilitates final prosthesis implantation.^[22]^

In this study, we determined the outcomes of patients underwent 2-staged THA after a course of antibiotic therapy.

## 2. Patients and methods

### 2.1. Study design and participants

This was a prospective observational investigation. We evaluated patients who were underwent 2-stage revision of a THA because of peri-prosthetic infection. Moreover, we assessed patients with revision surgery due to aseptic loosening. All patients had at least 15 months’ follow-up. We included patients by census-manner sampling and all eligible patients with sufficient data were enrolled.

### 2.2. Setting

During the study period, 51 revision THA procedures were performed by the senior author of the article (orthopedic surgeon) in Erfan hospital. From patients enrolled, 12 revisions were due to aseptic etiologies, and the left for a periprosthetic hip infection (loosening). Two stage revision surgery was performed for infected joints. Therefore, subjects were first underwent removal surgery of components and temporary antibiotic spacer were placed and simultaneously intravenous antibiotic were administered for at least 6 weeks. Then, reimplant was done if signs of infection were not resumed. Infected re-implantation THA were performed at the authors’ center. In all subjects, antibiotic treatment during surgery and 24 hours after it was prescribed. Primary demographic data and history of diseases are listed in Table 1. All subjects were underwent operation by senior author by similar surgery method at first arthroplasty. After joint exposure, samples were achieved from femoral, capsular, acetabular tissues and other suspected regions and evaluated for microbiologic culture. Positive culture were not reported in none of re-implanted patients.

**Table 1:** Primary diagnoses and demographic variables.

Characteristics	
Gender (n, %)	Male (16, 31.3) Female (35, 68.6)
Age (mean ± SD), yr	69.23 ± 15.79
Primary diagnosis	
Osteoarthritis (n, %)	12, 23.5
Osteonecrosis (n, %)	7, 13.7
Developmental hip dysplasia (n, %)	13, 25.4
Trauma (n, %)	15, 29.4
Rheumatoid arthritis (n, %)	4, 7.8
BMI (mean ± SD), kg/m^2^	29.89 ± 4.56

BMI = body mass index, SD = standard deviation.

### 2.3. Definitions and outcomes

PHI was determined by the existence of at least 1 of the next clinical criteria:

1. A purulent sinus correlated with the joint;
2. Perioperative evidence of suppuration; or
3. Laboratory or pathologic findings of infection or isolation of pathogens from 2 exclusive samples of articular aspiration or other samples.^[23]^ We defined “Persistent infection” as the existence of PHI after first-stage operation. It was the primary outcome. “Re-infection” was defined as PHI which happened after the finishing of SEA and antibiotic treatment. It was the secondary outcome in this study. Re-infection of the 2-stage revision THA was determined as profound infection of the hip prosthesis after first operation which needs re-operation to be treated. If seromas or superficial infection were increased by the deeper layers or to the hip prosthesis, it was considered as failure.

### 2.4. Intervention

After finishing intravenous antibiotic therapy within time of cement spacer placement, all subjects were underwent re-implantation. Infectious disease consult was done for giving longer period of antibiotic treatment after re-implant operation. At the centers of this study, there was no indications for prescription of oral antibiotic post-THA operation. If in any cases, oral antibiotic was started postoperatively, it was resumed for at least 14 days empirically and based on probable positive culture.

After re-implant operation, we followed up subjects in 4 time points: 1 week, 3 weeks, 3 and 6 months and then every year for at least 2 years. In each visits, they were evaluated for history and physical examination for finding signs of erythema, pain or discharge and dehiscence and others. Moreover, AP and lateral radiographs were taken in each visits for component placement or radiolucency assessment. If there was any evidence of re-infection, complete sepsis workup panel were performed.

### 2.5. Ethics

All cases were asked to fill written informed consent for participation in the study. All steps of the study was in line with ethical committee of hospital and Helsinki declaration.

### 2.6. Statistics

Descriptive statistical analyses was used to show mean and standard deviation of quantitative variables. We used paired sample *t* test for comparing before and after operation factors. Linear regression test was applied in finding predictors and Kaplan–Meier test used to estimate survival rate at 5 years. SPSS (SPSS 19.0 for Windows; SPSS Inc., Chicago, IL) software was used in all analyses.

## 3. Results

Fifty-one patients was included in the study. Most of them were female (68.6%) and the mean age of them was 69.23 ± 15.79 years old. The mean of BMI was 29.89 ± 4.56. Other baseline data is listed in Table 1.

After 2-staged revision operations, 40 cases had no problem until follow-ups. Eleven cases had persistent infection (27.5%) by severe hip pain, high CRP levels and poor wound improvement which needed to change their cement. Two cases (5%) needed 3 times for changing the prosthesis. Re-infection rate was 3 of 51 cases. Of these cases, one needed to removal of implant and 2 others managed by antibiotic therapy. List of infecting microorganisms is shown in Table 2. Data of persistent infection subjects after the first stage of the operation is summarized in Table 3.

**Table 2:** Infecting microorganisms.

	Number	Percent
MRSA	4	7.8
Klebsiella pneumoniae	1	1.9
*Pseudomonas aeruginosa*	2	3.9
*E coli*	2	3.9
Gram positive cocci	2	3.9

*E coli* = *Escherichia coli*, MRSA = methicillin-resistant *Staphylococcus aureus*.

**Table 3:** Summary of persistent infection cases after the first stage of the procedure.

Case	Infecting organism	Antibiotic(s) in spacer	Treatment	Outcome
1	MRSA	Vancomycin + imipenem	Repeated 1st stage surgery	Infection eradicated, followed by 2nd stage
2	MRSA	Vancomycin + gentamicin	Repeated 1st stage surgery	Persistent infection, followed by repeated debridement without reimplantation
3	MRSA	Vancomycin	Repeated 1st stage surgery	Infection eradicated, followed by 2nd stage
4	MRSA	Vancomycin + imipenem	Repeated 1st stage surgery	Infection eradicated, followed by 2nd stage
5	*Klebsiella pneumoniae*	Vancomycin + gentamicin	Repeated 1st stage surgery	Infection eradicated, followed by 2nd stage
6	*Pseudomonas aeruginosa*	Vancomycin + gentamicin	Repeated 1st stage surgery	Persistent infection, followed by repeated debridement without reimplantation
7	*Pseudomonas aeruginosa*	Vancomycin	Repeated 1st stage surgery	Infection eradicated, followed by 2nd stage
8	*E coli*	Vancomycin + gentamicin	Repeated 1st stage surgery	Infection eradicated, followed by 2nd stage
9	*E coli*	Vancomycin + gentamicin	Repeated 1st stage surgery	Infection eradicated, followed by 2nd stage
10	Gram positive cocci	Vancomycin	Repeated 1st stage surgery	Infection eradicated, followed by 2nd stage
11	Gram positive cocci	Vancomycin	Repeated 1st stage surgery	Infection eradicated, followed by 2nd stage

*E coli* = *Escherichia coli*, MRSA = methicillin-resistant *Staphylococcus aureus*.

The success rate of infection treatment, which is determined as the elimination of infection, was 78.4% (40 of 51) after the first operation and antibiotic treatment. This increases to 92.1% (47 of 51) after more debridement and antibiotic therapy.

None of other subjects received debridement, revision operation or antibiotic therapy for PHI within the follow-up period. Therefore, 47 patients had no infection at the final follow-up. The mean preoperative hip score was 8.5 ± 2.1 (6.2–9.1) which was improved at last follow up to 15.2 ± 1.7 (13.3–16.4). There were no complications except infection as mentioned earlier until last follow up.

## 4. Discussion

In this investigation, we demonstrated that short-term results of 2-staged revision of infected hip prosthesis is a safe method with low complications and high rate of success. Despite of lowing the rate of infection risk after primary THA to about 1%,^[2,5,6,8]^ remarkable percent of patients could lead to re-infection after 2-staged re-implant surgery. Eradication of such infections is so hard and have a high cost about 100 dollars per each revision.^[24]^ So, finding easy, cost-effective and available approaches to increase outcomes of patients to decrease re-infection rate after 2 stage revision THA, is necessary. In our investigation, the success rate was nearly 92%, which was better or equal to other studies.^[3,25–31]^

Previous investigations have shown that the antibiotic release from ALCS is great and constant.^[32,33]^ The important question, regarding a spacer’s efficacy in the treatment of PJI is, whether a spacer can achieve a certain antibiotic concentration in vivo or not. Downes et al reports negligible antibiotic levels beyond the fifth postoperative day in an in vivo study.^[34]^ In a systematic review, the in vivo antibiotic concentrations were measured during the first week after spacer implantation (7 studies), on the day of spacer removal or both. It was demonstrated a large increase in the antibiotic concentration on the first day after implantation, and then a rapid decrease during the next days. Furthermore, the antibiotic concentration at 6 weeks to 4 months after spacer removal, although lower than during the first week, was always higher than the MIC for the causative bacteria.^[35]^ In another in vivo study, it was shown that antibiotic washout from ALCSs was at least 4 months by loading 3.6 g of tobramycin and 1 g of vancomycin per 40 g package of bone cement.^[32]^ The efficacy of a spacer as a local carrier of antibiotics is based on its ability to reach high concentrations of the antibiotic at the site of infection for a suitable period of time.^[36]^

In another study, McDonald and his colleagues evaluated 82 patient with PHI after SEA and showed that re-infection happened in 3 of 7 subjects with retained cement. They also reported only 8 re-infection within the other patients (75 subjects) with cement removal.^[26]^

Using antibiotic perioperatively in 2-stage revision arthroplasty has been negotiated for its duration, special agents, and their impact on special organisms (such as methicillin-resistant Staphylococcus aureus). The suitable duration of parental antibiotic treatment is a controversial topic.^[17,18]^

Hsieh and his colleagues showed that antibiotic washout from ALCSs, was abrupt and great and the antibiotics (vancomycin and aztreonam) retained their therapeutic ability for several months.^[33]^ This success rate can be achieved by radical debridement. In a study of 31 consecutive infected THAs, McKenna et al^[18]^ explained that there was no recurrence in infection. They indicated that shortening postoperative antibiotic therapy leaded in infection treatment. Bassetti et al^[16]^ evaluated the duration of parenteral versus oral antibiotic (linezolid). They showed that both of them were effective in orthopedic procedures. Moreover, Oussedik et al^[19]^ demonstrated that linezolid is effective for the oral treatment of infected joint arthroplasties.

The optimal time of the re-implant operation is yet controversial. Prior investigations have suggested different intervals between steps, ranged from 1 month to 1 year.^[26,27]^ It is sometimes preferred to monitor the CRP level as an index of the response to treatment. This way seems to be more practical method than continue the fixed interval. Despite of not being specific to infection the CRP measurement, it is sensitive, accessible and most valuable when serially monitored.^[37]^ The use of a shortened antibiotic therapy between stages is not new.^[38,39]^ Stockley et al^[39]^ reported a success rate of 87.7% (100 of 114) in infection control with depot antibiotic treatment in the absence of long-term antibiotic therapy in PHI. Nelson et al,^[25]^ in a study of 28 patients who had an infection at the site of a hip or knee arthroplasty, reported that patients who were managed with the implantation of antibiotic cement beads in conjunction with <5 days of parenteral antibiotic therapy, and those who were managed with 6 weeks of intravenous antibiotic therapy, had comparable results.

## 5. Conclusion

Data from our prospective study showed that short-term antibiotic therapy in SEA could increase the rate of treatment failure in patients with PHI. Using effective staged surgical debridement, spacer of cement-antibiotic usage with monitoring erythrocyte sedimentation rate and CRP levels and performing re-implant operation with shortened IV antibiotic treatment, has led to better outcomes in this series of revised chronic hip joint prosthetic infection. Our results suggested that the routine usage of protracted antibiotic treatment courses might be beneficial. Performing large-scale, prospective, longer follow-up and randomized clinical trial to approve the findings is recommended.

## Author contributions

**Conceptualization:** Afshin Taheriazam.

**Data curation:** Amin Saeidinia.

**Investigation:** Afshin Taheriazam, Amin Saeidinia.

**Project administration:** Afshin Taheriazam.

**Writing – original draft:** Afshin Taheriazam, Amin Saeidinia.

**Writing – review & editing:** Afshin Taheriazam, Amin Saeidinia.
